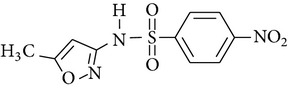# Oxidation of Sulfonamides in Aqueous Solution by UV-TiO_2_-Fe(VI)

**DOI:** 10.1155/2015/973942

**Published:** 2015-08-11

**Authors:** Yan Ma, Kejia Zhang, Cong Li, Tuqiao Zhang, Naiyun Gao

**Affiliations:** ^1^State Key Laboratory of Pollution Control and Resource Reuse, Tongji University, Shanghai 200092, China; ^2^Shanghai Urban Water Resources Development and Utilization National Engineering Center Co. Ltd., Shanghai 200082, China; ^3^College of Civil Engineering and Architecture, Zhejiang University, Hangzhou 310058, China; ^4^Key Laboratory of Drinking Water Safety and Distribution Technology of Zhejiang Province, Zhejiang University, Hangzhou 310058, China

## Abstract

The photocatalytic degradation of sulfonamides in aqueous TiO_2_ suspension under UV irradiation has been investigated using potassium ferrate as electron acceptors. The results showed that the stability of Fe(VI) is dependent on pH significantly, and the stability reduces obviously in the presence of UV-TiO_2_. The experiments indicated that Fe(VI) could effectively scavenge the conduction band electrons from the surface of TiO_2_. The photocatalytic oxidation of sulfonamides with Fe(VI) was found to be much faster than that without Fe(VI). The SD, SM, and SMX concentration was greatly reduced by 89.2%, 83.4%, and 82.0%, respectively, after 10 min with UV-TiO_2_-Fe(VI), comparing to 65.2%, 66.0%, and 71.9%, respectively, with Fe(VI) only in the dark and 71.3%, 72.7%, and 76.0%, respectively, with UV-TiO_2_. The pH value of solution significantly influenced the sulfonamides degradation in UV-TiO_2_-Fe(VI) system. The degradation amount of sulfonamides after 10 min was a maximum at pH 7. The intermediate products of sulfonamides oxidation by UV-TiO_2_-Fe(VI) were analysed by LC-HESI-MS-MS and the results suggested that a majority of sulfonamides turned into large-molecule products without complete mineralization.

## 1. Introduction

The widespread detection of pharmaceuticals active compounds in aquatic environments, which are now recognized as novel pollutants, is raising public health concerns due to the possibility of increased bacterial resistance [1]. Sulfonamides, also known as sulfa drugs, represent a kind of typical antibiotics and have been widely used in human and veterinary medicine to treat and prevent infectious bacterial diseases [2]. These sulfonamides are discharged into aquatic environment in their original or metabolized form mainly via disposal of expired pharmaceuticals, domestic wastewater effluents, and excretion [3]. It has been reported that sulfonamides are present in the concentration ranging from 0.13 to 1.9 *μ*g l^−1^ in aquatic environment [4]. Although sulfonamides are present in low concentrations, their existence in the environment may cause ecotoxicological effects [5]. The water contaminated by antibiotics is incompatible with conventional water and wastewater treatment methods [6]. Thus, it is necessary to develop more effective treatment technologies to remove sulfonamides from water.

Potassium ferrate (VI) (K_2_FeO_4_) is well known for a long time for its strong oxidizing power in acidic (*E* ^0^ = +2.20 V) and basic (*E* ^0^ = +0.72 V) solution [7] and for producing a coagulant (Fe(OH)_3_) from its reduced form. The previous studies indicate that the ferrate oxidation reaction from Fe(VI) to Fe(III) includes two sequential intermediates of Fe(IV) and Fe(V). The studies demonstrated that the reactivity of Fe(V) with compounds is 10^3^–10^5^ times more than Fe(VI) [8, 9], which means that the reduction from Fe(VI) to Fe(V) is a critical rate-determining step in the whole reaction and the oxidation efficiency of potassium ferrate can be enhanced by one-electron reducing agents, such as the conduction band electrons (e_cb_^−^) [10].

Photocatalytic water splitting on titanium dioxide (TiO_2_) was first discovered in 1972 [11]. Since then, a lot of attention has been paid to photocatalytic degradation of numerous organic contaminants in water using titanium dioxide in aqueous suspension, because of the strong oxidizing power of the photogenerated holes (h_vb_^+^) of TiO_2_ [12]. In the photocatalytic process, inhibiting the e_cb_^−^/h_vb_^+^ recombination by adding other electron acceptors to the reaction is one strategy to enhance oxidative efficiency [13]. The e_cb_^−^ is a good reductant and Fe(VI) is a strong oxidizing agent. Thus, the photoreduction of Fe(VI) may take place through one-electron steps that would result in the formation of Fe(V), Fe(IV), and Fe(III). Consider

(1) FeVI⁡⟶ecb−FeVFeV⟶ecb−FeIVFeIV⟶ecb−FeIII

In this study, potassium ferrate was used as electron acceptors to capture the electrons from TiO_2_ photocatalysis to form Fe(V), while inhibiting the e_cb_^−^/h_vb_^+^ recombination during photocatalytic reaction. Fe(VI) reduction and sulfonamides degradation in aqueous solution were studied under different conditions. This paper studied the analysis of the intermediate products and the pathways of sulfonamides degradation in the UV-TiO_2_-Fe(VI) system.

## 2. Materials and Methods

### 2.1. Materials

All chemicals employed in the laboratory experiments were purchased as analytical grade and used without any purification. The main chemical including potassium ferrate (>90% purity) and sulfonamides including sulfadiazine (SD), sulfamerazine (SM), and sulfamethoxazole (SMX) (>99%) were purchased from Sigma-Aldrich. The solutions were prepared with Milli-Q water. The Fe(VI) solutions were prepared by adding solid potassium ferrate to 0.001 mol l^−1^ borate/0.005 mol l^−1^ Na_2_HPO_4_ at pH 9.2 for the stability of ferrate solution [9]. The stock solutions of SD, SM, and SMX were prepared at concentration of 2 mmol l^−1^ in 0.01 mol·l^−1^ NaOH.  Table 1 shows the structure and the physicochemical properties of sulfonamides.

### 2.2. Methods

#### 2.2.1. Experimental Procedure

The stability of potassium ferrate in aqueous solution with different pH values from 7.0 to 11.3 was determined. The buffer solutions were prepared from K_2_HPO_4_, KH_2_PO_4_, and K_2_B_4_O_7_·5H_2_O with Milli-Q water. Each experiment, the Fe(VI) solution with an initial concentration of 0.2 mmol l^−1^, was prepared by adding a given quantity of solid K_2_FeO_4_ to the respective buffer solution, and the decomposition of samples was observed by determining the concentration of Fe(VI) at different time intervals.

The reaction solutions were prepared in 0.01 mol l^−1^ buffers to obtain the desired pH values. All experiments were carried out in 250 ml beakers at room temperature (25°C ± 2°C). Each experiment lasted for 10 min and samples were collected at different time intervals for SD, SM, and SMX analyses. A UV lamp (Philips) with a main emission at 254 nm was employed in this study. The light intensity on to the reaction solution was determined to be 0.15 mW cm^−2^. TiO_2_ (anatase, nanometre grade, <50 nm, BET 80–100 m^2^·g^−1^, Aladdin) was used as a photocatalyst. The TiO_2_ catalyst (500 mg l^−1^) and potassium ferrate were applied at different concentrations for the different experiments. Sodium hyposulfite solution was added immediately to the sample at each sampling time to stop any further reaction.

#### 2.2.2. Analytical Methods

The concentrations of potassium ferrate in aqueous solutions were determined by UV-vis spectroscopy (Unico WFZ UV-4802H). K_2_FeO_4_ dissolved as FeO_4_^2−^ has the absorption peak at 510 nm, and its molar absorptivity at 510 nm has been determined as 1150 M^−1^ cm^−1^ [14].

The concentration of SD, SM, and SMX was determined by HPLC (Waters e2695 Separation Module, Waters 2489 UV/visible detector), with a Waters bridge C18 column (150 mm × 4.6 mm) and ultraviolet detector setting wavelength of 270 nm at 35°C. Elution was performed with a mobile phase composed of acetonitrile/water with 0.1% formic acid (40/60, v/v) at a flow rate of 0.8 mL min^−1^.

Liquid chromatography (Waters e2695 Separation Module) together with heated electrospray ionization mass spectrometry (Thermo Finnigan TSQ Quantum) was used to detect the intermediate products of sulfonamides degradation. In the LC-HESI-MS-MS analysis, sample separation was conducted on a Thermo Basic C18 column (150 mm × 2.1 mm) at a flow rate of 0.3 ml min^−1^ and column temperature of 35°C. Chromatographic analyses were carried out using gradient elution with eluent A (acetonitrile) and eluent B (water with 0.1% formic acid). The analysis started with 10% of eluent A, held for 5 min, and then was increased linearly up to 40% in 15 min. This composition was returned to 10% of eluent A in 3 min, followed by a reequilibration time of 3 min, to give a total run time of 26 min. The ESI source was set in positive ion detection mode. The MS conditions were as follows: the spray voltage, 3.5 kV; sheath gas pressure, 40 psi; auxiliary gas pressure, 10 psi; capillary temperature 270°C, and the mass range is 50–500 *m*/*z*.

In this study, total organic carbon (TOC) of samples was determined by TOC analyzer (Shimadzu TOC-VCPH).

## 3. Results and Discussions

### 3.1. Stability of Potassium Ferrate

#### 3.1.1. The Effect of Solution pH

**Figure 1:**
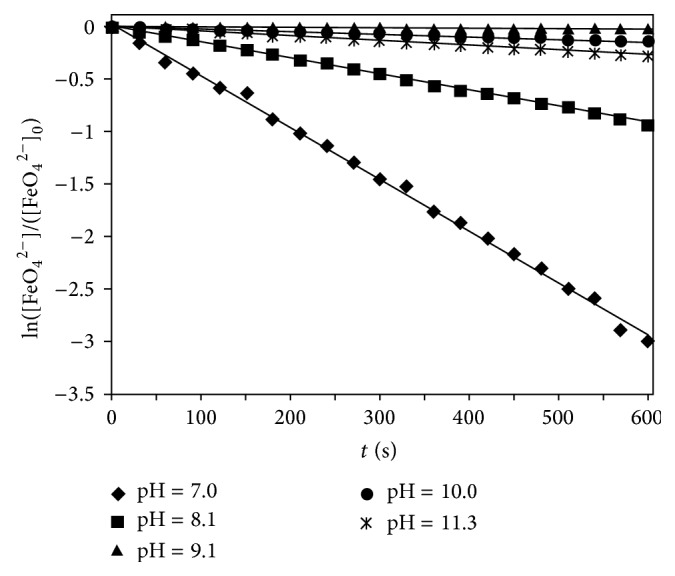
The decomposition of Fe(VI) in aqueous solution ([Fe(VI)]_0_ = 0.2 mmol l^−1^).

Aqueous potassium ferrate solutions with an initial concentration of 0.2 mmol l^−1^ were prepared with different pH values from 7.0 to 11.3. The Fe(VI) concentration was determined by UV-vis spectroscopy every 30 s. As shown in Figure 1, it is clear that the decomposition of Fe(VI) can be described with first-order kinetic model expressed by the following equation: *d*[FeO_4_^2−^]/*dt* = *k*[FeO_4_^2−^].   *R* ^2^ values which are greater than 0.99 for all pH values (Table 2) also show the applicability of this model to describe ferrate decomposition.

The decomposition kinetic constants are shown in Table 2, indicating that the stability of the ferrate is highly pH dependent, and Fe(VI) is more stable in alkaline conditions. It is evident that there appeared to be a maximum stability at pH 9.0–10.0 and Fe(VI) is highly unstable at pH <7.

#### 3.1.2. The Effect of UV-TiO_2_

**Figure 2:**
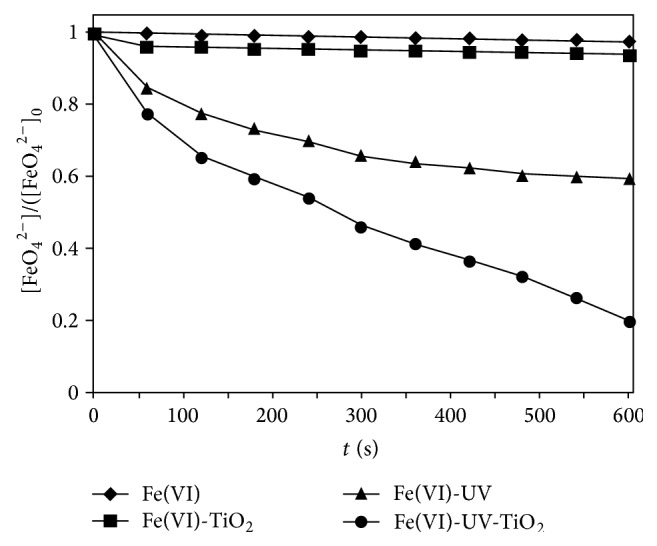
The decomposition of Fe(VI) in aqueous solution under UV irradiation ([Fe(VI)]_0_ = 0.2 mmol l^−1^, light intensity = 0.15 mW cm^−2^, pH 9.1, and [TiO_2_] = 500 mg l^−1^).

Aqueous potassium ferrate solutions (0.2 mmol l^−1^) were prepared at pH 9.1. The decomposition of potassium ferrate in aqueous TiO_2_ suspension under UV irradiation is shown in Figure 2. At high pH (9.1), potassium ferrate was very stable, and adding TiO_2_ had little effect on the stability of potassium ferrate without UV irradiation. Under UV irradiation, the stability of potassium ferrate could be decreased significantly. The decomposition rate of potassium ferrate was the highest in aqueous Fe(VI) + TiO_2_ solution under UV irradiation, and about 80% of potassium ferrate was decomposed after 10 min of photocatalytic reaction. In UV-TiO_2_-Fe(VI) system, Fe(VI) captured the electrons from TiO_2_ to form Fe(V), Fe(IV), and Fe(III) through one-electron steps, which accelerated the decomposition rate of potassium ferrate.

### 3.2. Degradation of Sulfonamides in UV-TiO_2_-Fe(VI) System

#### 3.2.1. Sulfonamides Degradation

**Figure 3:**
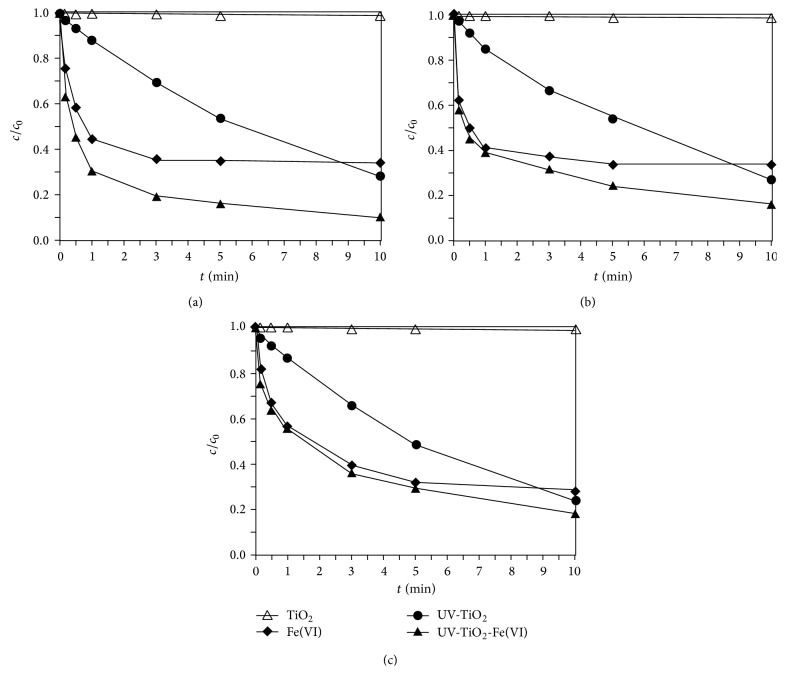
The removal of different sulfonamides by UV-TiO_2_-Fe(VI) ((a) sulfadiazine; (b) sulfamerazine; (c) sulfamethoxazole, [sulfadiazine]_0_ = 0.02 mmol l^−1^, [sulfamerazine]_0_ = 0.02 mmol l^−1^, [sulfamethoxazole]_0_ = 0.02 mmol l^−1^, [Fe(VI)]_0_ = 0.05 mmol l^−1^, pH 7, [TiO_2_] = 500 mg l^−1^, and light intensity = 0.15 mW cm^−2^).

In order to analyse the degradation of sulfonamides at different conditions, a set of experiments was carried out under four conditions: (I) TiO_2_ only in the dark, (II) Fe(VI) only in the dark, (III) UV-TiO_2_, and (IV) UV-TiO_2_-Fe(VI). The experimental results are shown in Figure 3. Figure 3 shows that the final concentrations of sulfonamides (SD, SM, and SMX) were almost the same with the initial concentration, verifying that no losses occurred from TiO_2_ only in the dark. The degradation of SD, SM, and SMX by ferrate oxidation only after 10 min reaction was achieved by 65.2%, 66.0%, and 71.9%, respectively; and the SD, SM, and SMX degradation by catalytic oxidation alone (UV-TiO_2_) was achieved by 71.3%, 72.7%, and 76.0%, respectively. Under UV irradiation together with TiO_2_ and Fe(VI), the concentrations of SD, SM, and SMX were greatly reduced by 89.2%, 83.4%, and 82.0%, respectively, after 10 min. Due to the interaction of photocatalytic oxidation and Fe(VI) oxidation, higher rate of sulfonamides degradation was achieved, and the oxidation of sulfonamides was enhanced greatly in the UV-TiO_2_-Fe(VI) system. In this interactive reaction, Fe(VI) captured the e_cb_^−^ from TiO_2_ to form Fe(V), which could inhibit the recombination of e_cb_^−^/h_vb_^+^ simultaneously [10, 13]; sulfonamides were quickly degraded by several oxidants including ^•^OH, h_vb_^+^, Fe(VI), and Fe(V). The previous study [8] demonstrated that the reaction rate of Fe(V) with compounds is 3–5 orders of magnitude faster than Fe(VI). As a result, the degradation of sulfonamides in the UV-TiO_2_-Fe(VI) system could be accelerated significantly.

#### 3.2.2. The Effect of Solution pH

**Figure 4:**
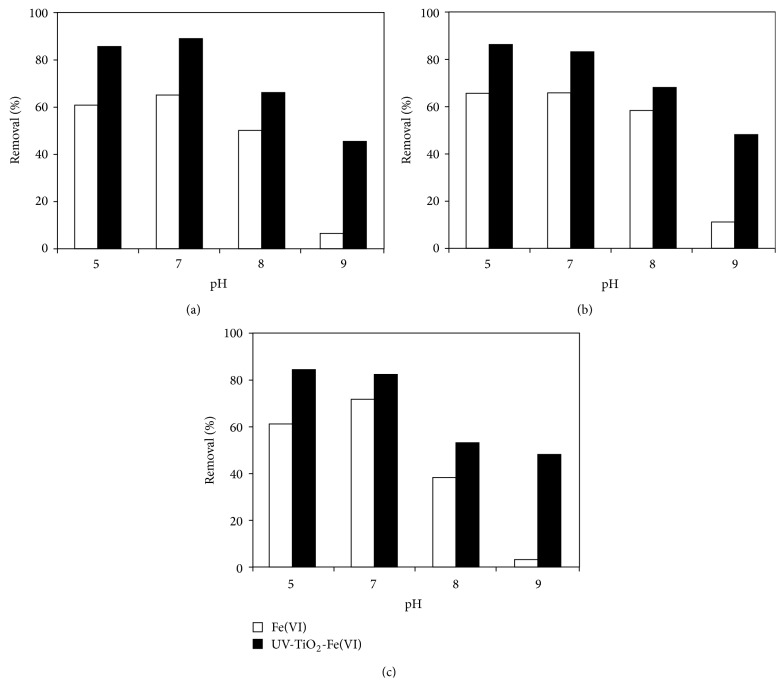
The removal of different sulfa antibiotics by UV-TiO_2_-Fe(VI) under different pH values ((a) sulfadiazine; (b) sulfamerazine; (c) sulfamethoxazole, [sulfadiazine]_0_ = 0.02 mmol l^−1^, [sulfamerazine]_0_ = 0.02 mmol l^−1^, [sulfamethoxazole]_0_ = 0.02 mmol l^−1^, [Fe(VI)]_0_ = 0.05 mmol l^−1^, [TiO_2_] = 500 mg l^−1^, and light intensity = 0.15 mW cm^−2^).

**Figure 5:**
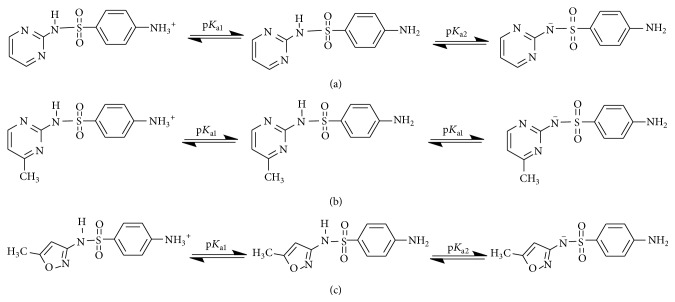
The protonation and deprotonation of sulfonamides ((a) sulfadiazine; (b) sulfamerazine; (c) sulfamethoxazole).

The experiments were performed in the pH range of 5–9. As shown in Figure 4, the removal of sulfonamides is much higher in UV-TiO_2_-Fe(VI) system than that resulting from ferrate oxidation alone in the pH range of 5–9, and the solution pH value significantly influenced the sulfonamides degradation. At pH 7, the removal of SD, SM, and SMX is the highest in the pH range of 5–9. The possible reason for the increased degradation is that this pH (7) is close to the pKa_2_ values of SD (6.5), SM (7.0), and SMX (5.7). At this pH, SD, SM, and SMX are dissociated (Figure 5). Previous studies found that the dissociation of the compound increases with increasing pH and deprotonated compounds are more readily oxidized by potassium ferrate and other oxidants such as ^•^OH and h_vb_^+^ [15, 16]. Meanwhile, potassium ferrate had a much higher oxidation potential at acidic condition (*E* ^0^ = 2.20 V) than that at basic conditions (*E* ^0^ = 0.72 V) [17]. At pH <7, although oxidative ability of ferrate is high, the ferrate is highly unstable (Figure 1). Therefore, at pH 5, most Fe(VI) is decomposed to make the removal rate of sulfonamides low. In the UV-TiO_2_-Fe(VI) system, the ferrate oxidations of sulfonamides were enhanced most significantly at pH 9 due to the low oxidation ability of ferrate.

#### 3.2.3. Pathways of Sulfonamides Degradation with UV-TiO_2_-Fe(VI)

**Figure 6:**
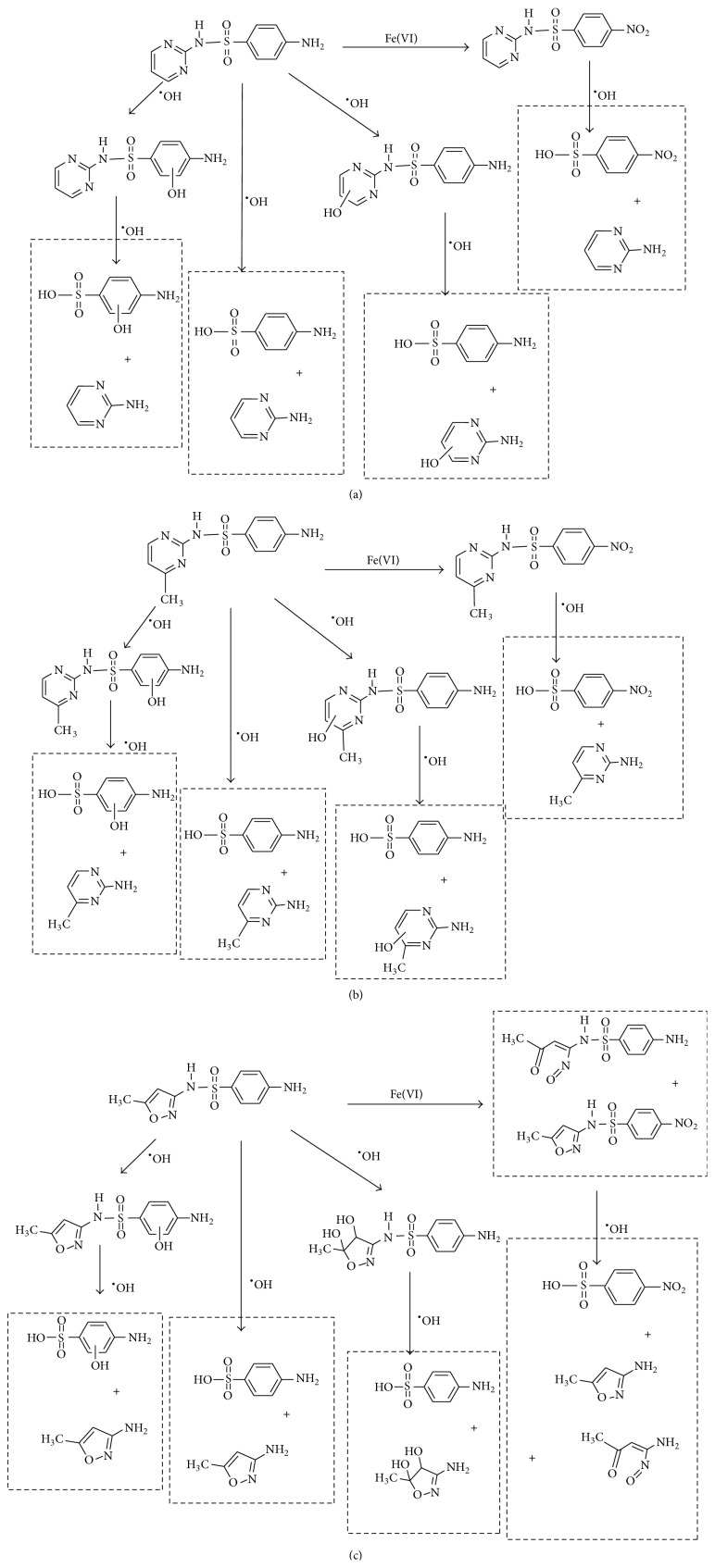
Proposed degradation pathway of sulfa antibiotics oxidized by potassium ferrate combined with photocatalytic oxidation ((a) sulfadiazine; (b) sulfamerazine; (c) sulfamethoxazole).

The formation of intermediates products was discussed in the experiments, in which sulfonamides were degraded in the UV-TiO_2_-Fe(VI) system. The samples were taken after 10 min of reaction and analysed by LC-HESI-MS-MS. The main identified intermediates of sulfonamides are shown in Table 3. The *m*/*z* values correspond to [M + 1]^+^ ions in the positive mode of LC-HESI-MS-MS. According to the results, the molecular structures of SD, SM, and SMX have molecular weights [M + 1]^+^ = 251, 265, and 254. Four intermediates have been identified for SD and SM, whose molecular weights were 267, 173, 96, and 281 and 281, 173, 110, and 295, respectively. Five intermediates have been identified for SMX, whose molecular weights were 270, 288, 173, 99, and 284. The peak corresponding to the molecular weights 267, 281, and 270 seems to be hydroxylated analogues of SD, SM, and SMX. As shown in Table 3, it seems that the N–S in sulfonamides can be cleaved by oxidation of UV-TiO_2_-Fe(VI). The attack on NH_2_ group of the aniline moiety as well as the isoxazole moiety of SMX happened during the ferrate oxidation, involving a single electron-transfer mechanism as shown by Sharma [18] and Huang [19]. Based on these identified intermediate products, the pathways for the sulfonamides degradation by UV-TiO_2_-Fe(VI) are proposed schematically in Figure 6. The results indicated that a majority of sulfonamides transformed into large-molecule products without complete mineralization. Total organic carbon analysis was performed to observe the mineralization efficiency of sulfonamides degraded by UV-TiO_2_-Fe(VI). The initial TOC of SD, SM, and SMX was 2.185 mg·l^−1^, 2.267 mg·l^−1^, and 2.194 mg·l^−1^, respectively, and the TOC of SD, SM, and SMX after 10 min reaction was 2.172 mg·l^−1^, 2.255 mg·l^−1^, and 2.181 mg·l^−1^, respectively, indicating that the degradation of sulfonamides mostly produced intermediate products and little mineralization to carbon dioxide in the UV-TiO_2_-Fe(VI) system.

## 4. Conclusions

In this study, sulfonamides as typical antibiotic chemicals were studied to be degraded in the UV-TiO_2_-Fe(VI) system. The experimental results showed that the decomposition rate of Fe(VI) was highly dependent on pH, and the stability of Fe(VI) reduced obviously in the presence of UV-TiO_2_. The results also indirectly demonstrated that Fe(VI) could be reduced by e_cb_^−^ on the TiO_2_ surface to form Fe(V) and to inhibit e_cb_^−^/h_vb_^+^ recombination in the UV-TiO_2_-Fe(VI) system, which can significantly enhance the removal of sulfonamides. Therefore, the combination of photocatalytic oxidation and ferrate oxidation is an effective treatment technology for the treatment of sulfonamides in aquatic environment. In order to identify the formation of intermediate reaction products and clarify the degradation pathways of sulfonamides in the UV-TiO_2_-Fe(VI) system, the extension of sulfonamides degradation and TOC mineralization were monitored in this study. The analyses by LC-HESI-MS-MS and TOC analyzer indicated that a majority of sulfonamides turned into large-molecule products without complete mineralization.

## Figures and Tables

**Table 1:** The physical and chemical properties of sulfonamides standards.

Chemical name	Molecular formula	Chemical structure	Molecular weight/Da.	p*K*_a_ [20]	
				p*K*_a1_	p*K*_a2_
Sulfadiazine	C_10_H_10_N_4_O_2_S	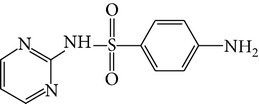	250.27	2.49	6.50

Sulfamerazine	C_11_H_12_N_4_O_2_S	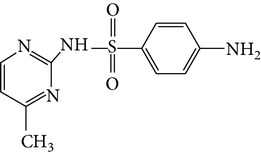	264.30	—	7.00

Sulfamethoxazole	C_10_H_11_N_3_O_3_S	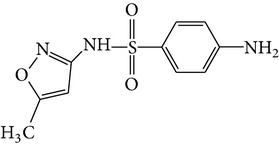	253.28	1.74	5.70

**Table 2:** The kinetic constants of Fe(VI) decomposition with pH.

pH	7.0	8.1	9.1	10.0	11.3
*k* (×10^−4^ s^−1^)	49.1	15.5	0.367	2.27	4.65
*R* ^2^	0.9975	0.9987	0.9972	0.9981	0.9988

**Table 3:** The main identified intermediates of sulfonamides.

Chemical name	*m*/*z*	Molecular structure
Sulfadiazine	267	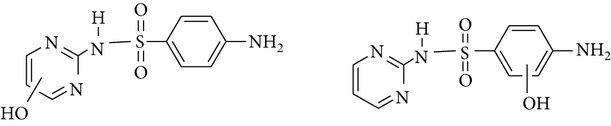
	173	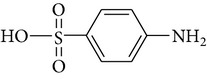
	96	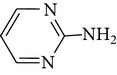
	281	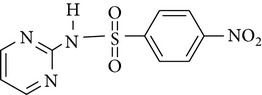

Sulfamerazine	281	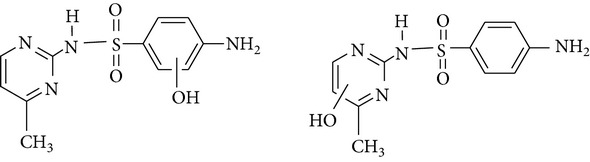
	173	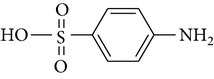
	110	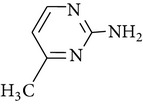
	295	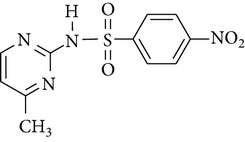

Sulfamethoxazole	270	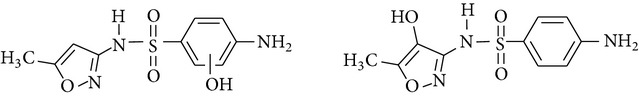
		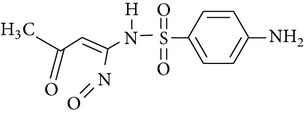
	288	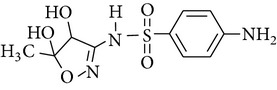
	173	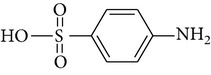
	99	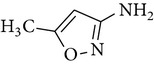
	284